# Recurrence Risk Factors Analysis for Stage I Non-small Cell Lung Cancer

**DOI:** 10.1097/MD.0000000000001337

**Published:** 2015-08-14

**Authors:** Ching-Feng Wu, Jui-Ying Fu, Chi-Ju Yeh, Yun-Hen Liu, Ming-Ju Hsieh, Yi-Cheng Wu, Ching-Yang Wu, Ying-Huang Tsai, Wen-Chi Chou

**Affiliations:** From the Division of Thoracic and Cardiovascular Surgery, Department of Surgery (C-FW, Y-HL, M-JH, Y-CW, C-YW); Division of Pulmonary and Critical Care, Department of Internal Medicine (J-YF); Division of Pathology, Chang Gung Memorial Hospital, Taoyuan (C-JY); Division of Pulmonary and Critical Care, Department of Internal Medicine, Chang Gung Memorial Hospital, Chiayi (Y-HT); and Division of Oncology, Department of Internal Medicine, Chang Gung Memorial Hospital, Taoyuan, Taiwan (W-CC).

## Abstract

Lung cancer is the leading cause of cancer-related death worldwide. Even early-stage patients might encounter disease recurrence with relative high risk. Effective postoperative therapy is based on an accurate assessment of treatment failure after surgery. The aim of this study is to construct a disease-free survival (DFS) prediction model and stratify patients into different risk score groups.

A total of 356 pathological stage I patients (7th American Joint Committee on Cancer) who underwent lung resection from January 2005 through June 2011 were retrospectively reviewed. Of these patients, 63 patients were eliminated for this study. A total of 293 p-stage I patients were included for further univariate and multivariate analysis. Clinical, surgical, and pathological factors associated with high risk of recurrence were analyzed, including age, gender, smoking status, additional primary malignancy (APM), operation method, histology, visceral pleural invasion, angiolymphatic invasion, tumor necrosis, and tumor size.

Of the 293 p-stage I non-small cell lung cancer (NSCLC) patients examined, 143 were female and 150 were male, with a mean age of 62.8-years old (range: 25–83-years old). The 5-year DFS and overall survival rates after surgery were 58.9% and 75.3%, respectively. On multivariate analysis, current smoker (hazards ratio [HR]: 1.63), APM (HR: 1.86), tumor size (HR: 1.54, 2.03), nonanatomic resections (HR: 1.81), adenocarcinoma histology (HR: 2.07), visceral pleural invasion (HR: 1.54), and angiolymphatic invasion (HR: 1.53) were found to be associated with a higher risk of tumor recurrence. The final model showed a fair discrimination ability (C-statistic = 0.68). According to the difference risk group, we found patients with intermediate or higher risk group had a higher distal relapse tendency as compared with low risk group (*P* = 0.016, odds ratio: 3.31, 95% confidence interval: 1.21–9.03).

Greater than 30% of disease recurrences occurred after surgery for stage I NSCLC patients. That is why we try to establish an effective DFS predicting model based on clinical, pathological, and surgical covariates. However, our initial results still need to be validated and refined into greater population for better application in clinical use.

## INTRODUCTION

Lung cancer is the main cause of cancer-related deaths worldwide.^1^ Surgery constitutes the primary therapeutic option for the management of early-stage non-small cell lung cancer (NSCLC).^2^ Even early-stage patients might encounter disease recurrence with relative high risk.^3,4^ Effective postoperative therapy is based on an accurate assessment of treatment failure after surgery. Recently, adjuvant cisplatin-based regimens have demonstrated survival benefits in early-stage NSCLC patients.^5,6^ In addition, it has been reported that oral administration of the combination drug uracil-tegafur (UFT) or S-1 improved overall survival and disease-free survival (DFS) of patients who underwent complete resection for stage I NSCLC adenocarcinoma.^7–9^ However, the risk factors of treatment failure after surgery have not been well described, and clinical pathologic features placing patients at particularly high risk of tumor recurrence have been inadequately studied. Only when a complete prediction model can be imposed on stage I NSCLC patients, can each patient receive adequate adjuvant therapy and follow-up program. The aim of this study is to construct a DFS prediction model and stratify patients into different risk groups.

## MATERIALS AND METHODS

### Patients

This study was approved by the institutional review board of the Chang Gung Memorial Hospital (IRB No: 103-5631B). Because this was a retrospective study, the need to obtain written informed consent from each patient was waived.

We performed a retrospective review of 356 patients who underwent lung resection for pathological stage I NSCLC (7th American Joint Committee on Cancer) in our prospective lung cancer database from January 2005 through June 2011. Sixty three patients were eliminated from this study, whereby exclusion criteria included an incomplete medical record, loss of patients to follow-up, surgical margin positive patients, or patients’ receiving neoadjuvant therapy or scheduled adjuvant therapy. Medical records and pertinent radiologic imaging were reviewed to characterize each patient's demographic information, obtain surgical and pathologic details, and record patterns of failure after surgery. The preoperative workup included chest radiography, bronchoscopy, chest computed tomography (CT), spirometry, bone scan, and a thorough search for distant metastases, such as positron emission tomography (PET) imaging and brain CT.

### Surgical Technique

Lobectomy, bilobectomy or pneumonectomy, and wedge resection with systemic lymphadenectomy were performed according to the patient's preoperative physiological condition and tumor location. At least 3 mediastinal nodes are excised as a minimum requirement. All of the resected lymph nodes were labeled separately. All pulmonary resections were performed by open thoracotomy (open) or video-assisted thoracoscopic surgery. Surgical resections included 2 pneumonectomies, 3 bilobectomies, 263 lobectomies, and 25 wedge resections.

### Pathological Evaluation

According to the TNM classification of the 7th American Joint Committee for Cancer Staging, all patients were staged as final pathologic stage I. The recorded pathological variables included tumor size, tumor differential grade, visceral pleural invasion,^10–12^ angiolymphatic invasion,^12–14^ tumor necrosis,^15^ tumor histology, and lymph node dissection numbers.^16^

### Follow-Up

After surgery, patients were examined on an outpatient basis at 3 to 6-month intervals. Chest radiography or chest CT was utilized for surveillance imaging. If disease relapse was suspected, brain magnetic resonance imaging or 18F-fluorodeoxyglucose PET was arranged. The main purpose of this study was to assess the risk of disease recurrence after surgery and to identify clinical, surgical, and pathological features which were associated with high recurrence risk. Local recurrence was defined as disease recurrence at the surgical resection margin, ipsilateral hilum, and/or mediastinum. All other sites of failure were considered distant recurrences.

### Statistical Analysis

SPSS (V17.0, SPSS, Inc, Chicago, IL) and STATA (version 12.1) were used for statistical analysis. DFS period was calculated from the date of surgery to the date of treatment failure (defined as local and/or distant recurrence). DFS curves were estimated using the Kaplan–Meier method. Significance was assessed using the log rank test. A *P* value of <0.05 was considered to indicate statistical significance. Possible predictors of DFS were investigated using Cox multivariate proportional hazards. The discriminative ability of prognostic models was evaluated by Harrell concordance index (C-index), which is a natural extension of the receiver-operating characteristic curve area to survival analysis and ranges from 0.5 (no discrimination) to 1 (perfect discrimination). The additive risk score was derived from regression model coefficients of the significant predictor: the smallest coefficient was given a value of 1 and the other values were assigned by the reason of proportional increasing.

## RESULTS

**FIGURE 1:**
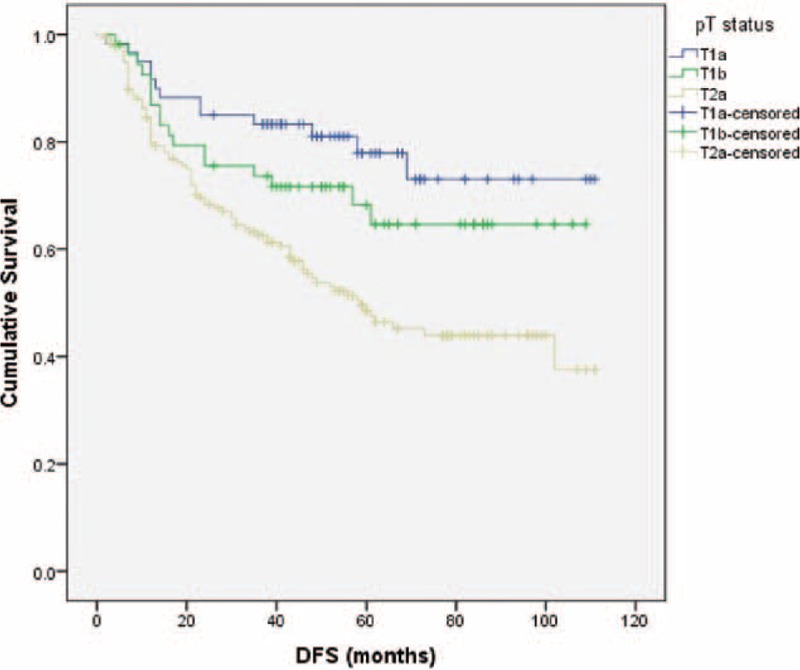
Disease-free survival rates of stage I patients with different pT status (*P* < 0.001).

Of the 293 p-stage I NSCLC patients examined, 143 were female and 150 were male, with a mean age of 62.83-years old (range: 25–83-years old). The mean follow-up time was 46 months. The majority of patients had stage Ib disease (38.6% with stage Ia and 61.4% with stage Ib). The average number of hilar lymph nodes retrieved was 7.45, and the average total number of lymph nodes dissected was 17.67. Surgical margins were all negative in 293 patients. Angiolymphatic invasion was seen in 66 patients (22.5%), and visceral pleural invasion was noted in 130 patients (44.3%). The characteristics of patients’ profiles are shown in Table 1. For all patients, the 5-year DFS and overall survival rates after surgery were 58.9% and 75.3%, respectively. When evaluated by T stage, patients with T1a tumors had 78% DFS, whereas patients with T1b and T2a tumors had an estimated 68.2% and 50.7% 5-year DFS rate (*P* < 0.001 Figure 1).

**TABLE 1:**
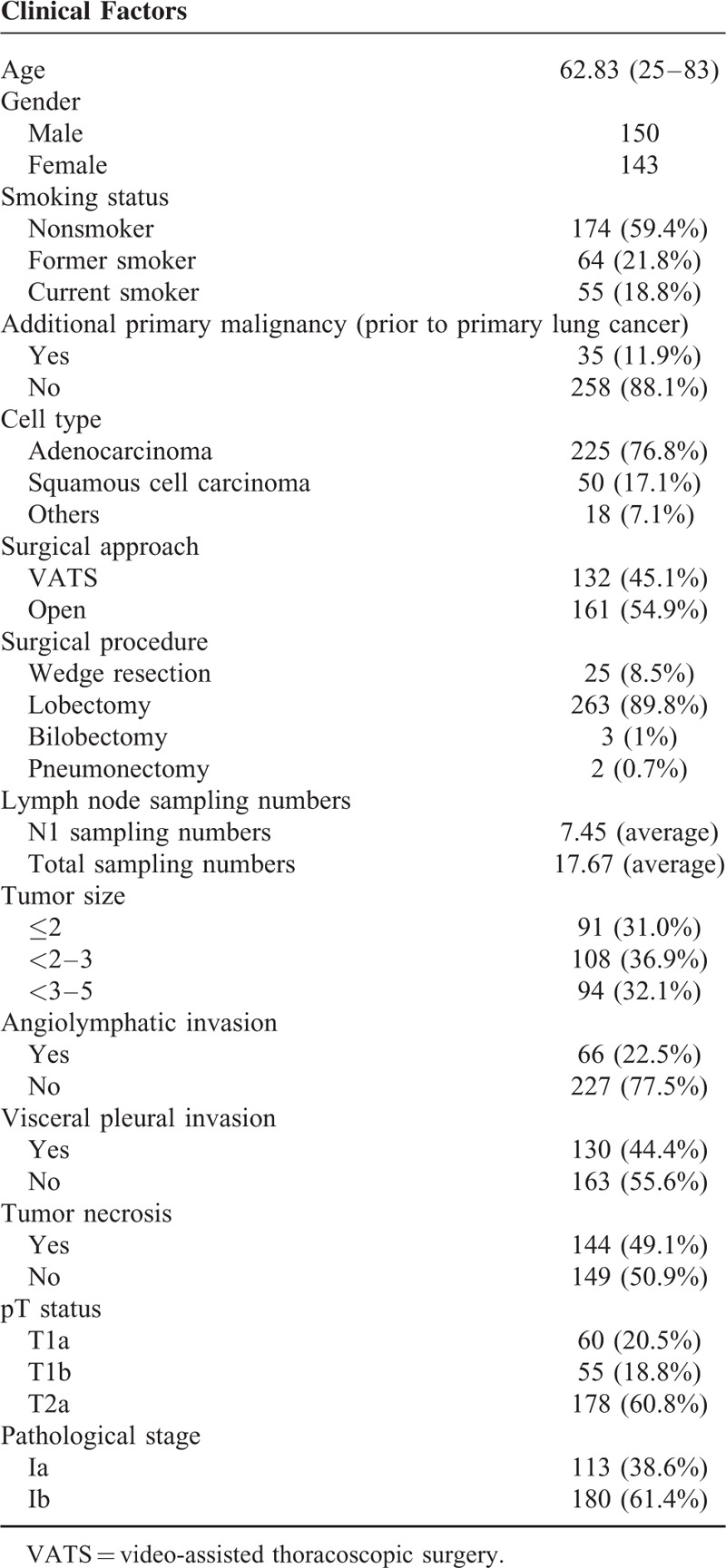
Patient Demographics and Characteristics the detection of APM is not uncommon in NSCLC patients^20^

Patients with previous history of malignancy have a poor DFS rate (*P* = 0.002). The 5-year DFS rates for adenocarcinoma and nonadenocarcinoma were 55.4% and 65.1%, respectively (*P* = 0.144). The 5-year DFS rates of patients with and without angiolymphatic invasion were 45% and 62.1%, respectively (*P* = 0.001). DFS was shown to be significantly longer in patients without visceral pleural invasion. These patients had an average 5-year DFS rate of 69.8%, in contrast to 46% in those with visceral pleural invasion (*P* < 0.001).

Disease recurrence was identified in 115 patients. Disease recurrence was confirmed by means of biopsy in 47% of patients, while the remaining 53% of patients were confirmed by means of CT or PET scan. If patients had previous history of malignancy, biopsy was conducted. Local recurrence was noted in 50 patients and distal recurrence in 65 patients. The median time from surgery to recurrence was 16 months for those patients with local recurrence and 18 months for those with distal recurrence.

**FIGURE 2:**
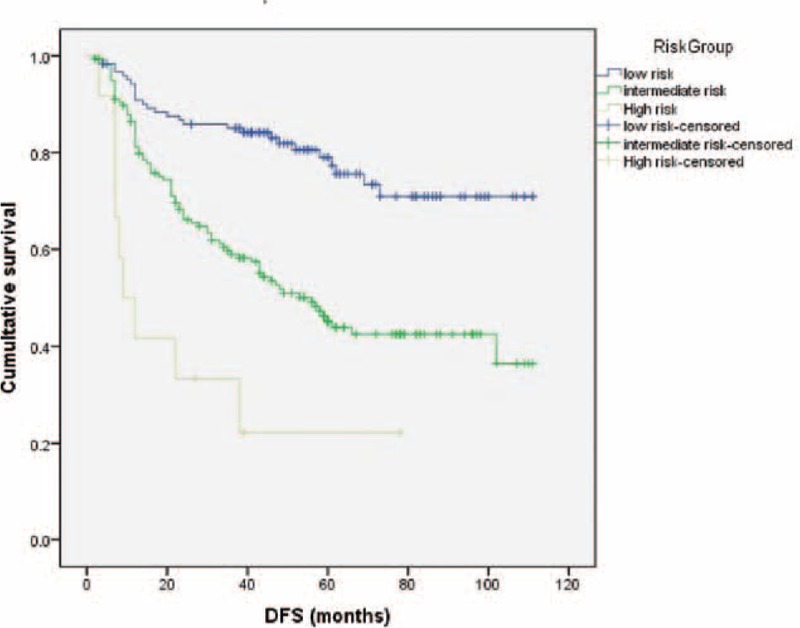
Disease-free survival rates for different risk group (*P* < 0.001).

**FIGURE 3:**
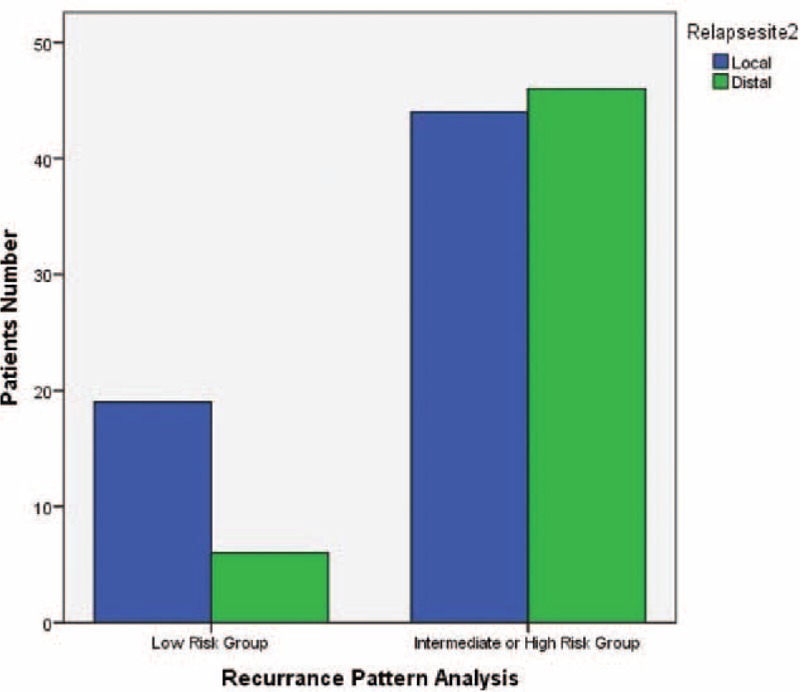
Relapse site analysis with risk score (*P* = 0.016, odds ratio: 3.31, 95% confidence interval [CI]: 1.21–9.03).

Variables associated with a higher rate of treatment failure on univariate analysis were wedge resection, previous history of malignancy, current smoker, tumor size, angiolymphatic invasion, and visceral pleural invasion. Detailed univariate analysis is listed in Table 2. For the multivariate analysis, possible prognostic factors associated with DFS were considered in a multivariable Cox proportional hazard regression analysis and presented in Table 3. Predictors of DFS associated with *P* ≤ 0.05 on multivariate analysis were included in the final score model. The additive risk score was designed from the hazard ratio of significant predictor estimates (Table 4). The resulting additive risk score identifies 3 groups, each presenting a different DFS curve (*P* < 0.001, Figure 2). We further analyzed the relationship between the relapse pattern and the risk group. We found patients with intermediate or higher risk group had a higher distal relapse tendency as compared with low risk group (*P* = 0.016, odds ratio: 3.31, 95% confidence interval: 1.21–9.03) (Figure 3).

**TABLE 2:**
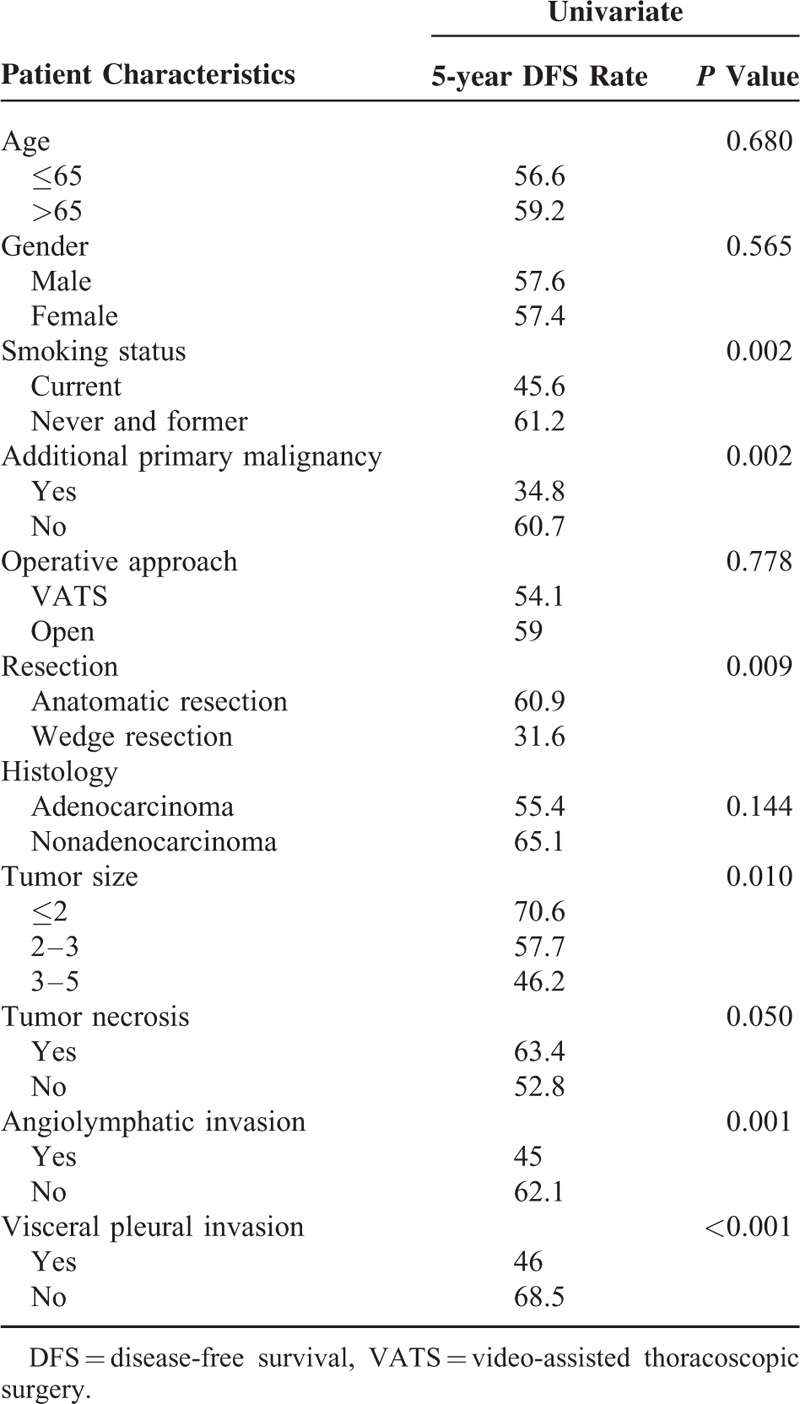
Clinical-Pathological Risk Factors Univariate Analysis

**TABLE 3:**
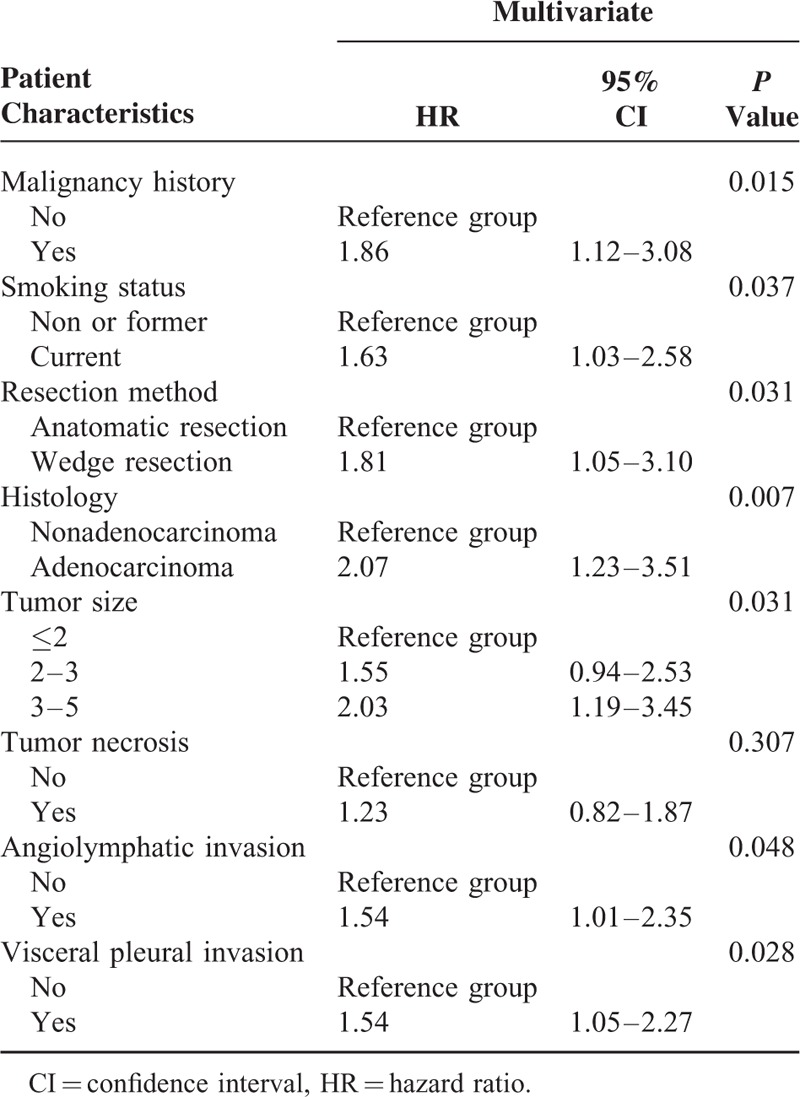
Clinical-Pathological Risk Factors Multivariate Analysis

**TABLE 4:**
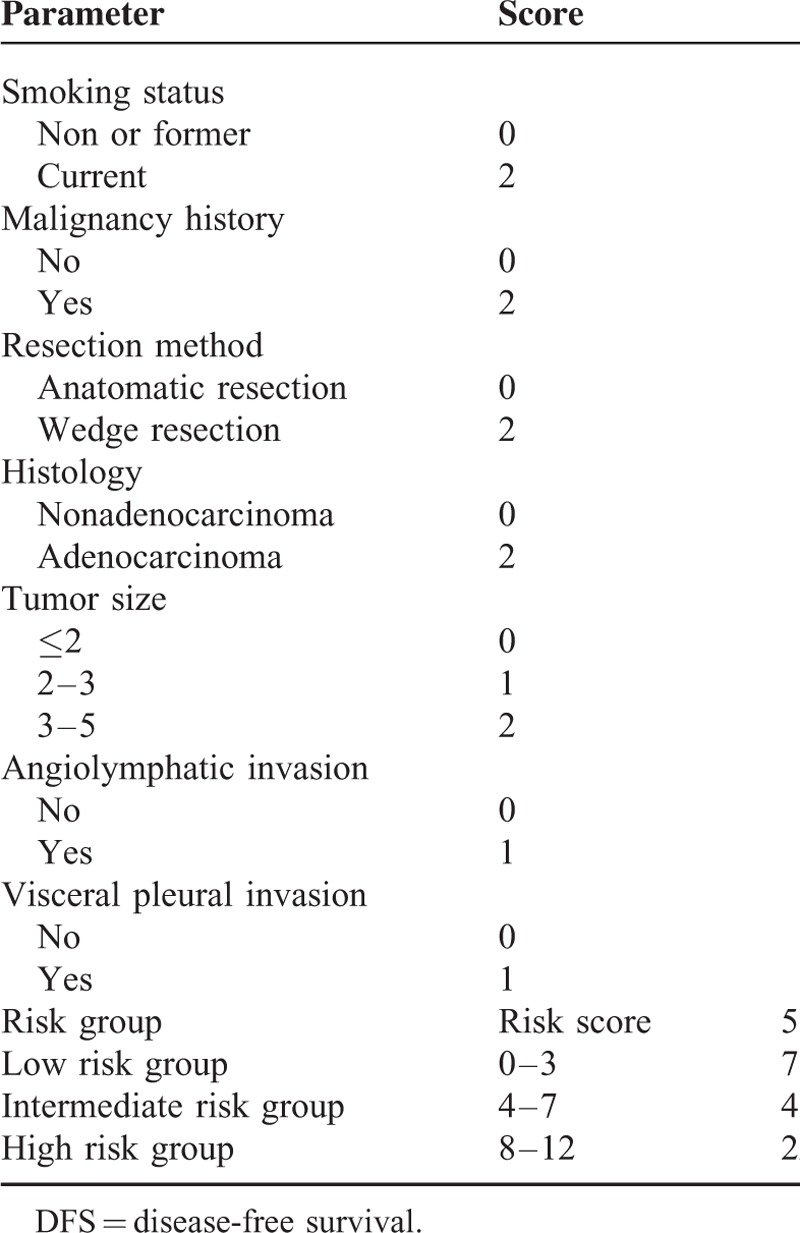
Risk Factor Calculation and Risk Group Stratification

## DISCUSSION

This study represents an attempt to create a prognostic model for clinical and pathological DFS in surgically resected stage I NSCLC patients. Correct prediction for patients with NSCLC is clinically essential but complex to define. The TNM-staging system has been the only validated prognostic indicator for survival,^1^ but there are reports of large variations, ranging from 55% to 85%.^17–19^ A more refined estimate of outcome in early-stage NSCLC is DFS. In order to clarify and stratify patients into different risk groups, we give each risk factor its own score by regression model coefficient and stratify them into 3 groups. We found that if patients belong to the low risk group, longer DFS could be expected (Figure 2). Moreover, in the lower risk score group (score: 0–3) there was a higher tendency of local recurrence compared with intermediate or higher risk group (Figure 3). Different follow-up program may be considered for low or intermediate and high risk group. Routinely chest X-ray may be insufficient for intermediate and high risk group patients. Further prospective study is needed.

Our study suggests that a history of additional primary malignancy (APM), current smoker, tumor size, angiolymphatic invasion, visceral pleural invasion, and nonanatomic resection (wedge resection) were all independent risk factors. Due to medical and surgical improvements, the detection of APM is not uncommon in NSCLC patients.^20^ The incidence rate of APM ranges from 8% to 12.8% in reports.^21^ Cho et al^22^ showed APM was a poor prognostic factor in stage I gastric cancer.^23^ Our study revealed similar findings in stage I NSCLC; however, due to the limited case numbers, the effect of APM warrants further investigation. DFS deteriorated with different smoking habits. Current smoker was a poor prognostic factor for DFS compared with nonsmoker and ex-smoker (*P* = 0.002). In addition, nonadenocarcinoma patients were found to have a better DFS rate in the multivariate analysis. Our result showed some discrepancy with several reports, in which showed that adenocarcinoma is at least equal or do better than squamous histology in patients with early-stage lung cancer.^3,4^ However, smoking habits might affect the survival result of different histology type of lung cancer. Michael et al^23^ reported that squamous histology was a significant beneficial factor compared with adenocarcinoma, with regard to survival.^24^ In his study, smoking status at time of surgery does not affect long-term survival in patients with squamous cell carcinoma, but make a significant difference to the long-term outcomes of patients with adenocarcinoma.^24^ Besides, from view of actual pharmacologic effect, quitting smoking showed different effect among different cell type of pulmonary malignancy.^25^ In our study, 55 current smokers were identified. A 75% belongs to adenocarcinoma patients and 25% belongs to nonadenocarcinoma patients. With regard to ex-smoker, 69% belongs to adenocarcinoma and 31% belongs to nonadenocarcinoma patients. Owing to limited numbers of patients, we did not put smoking habits and different histology-type lung cancer into further evaluation. In addition, although patients with squamous cell carcinoma constitute majority of nonadenocarcinoma population, different cell types of pulmonary malignancy, such as carcinoid tumor, mucoepidermoid tumor, were also included in this group. This might affect result in our analysis. In future, we may enroll more patients to validate the role of different histologies in terms of DFS and further analyze the survival impact of smoking habits on different cell type.

As with any retrospective analysis, the current study has limitations. Although nearly 50% of local recurrences were confirmed with biopsy, the remaining was scored using imaging studies that can lead to overestimation. In addition, our 5-year DFS rate is inferior to that of a Japanese report, especially with regard to pIb patients.^26,27^ This could be explained in part by the routine use of UFT or S-1 as adjuvant therapy for stage I adenocarcinoma patients in Japan^7–9^ and the fact that plb patients constitute the majority of stage I patients in our study (61.4%). Our national health insurance began to cover UFT usage in pIb adenocarcinoma patients in 2010. In the current study, we excluded patients who received UFT therapy as scheduled adjuvant therapy. In future, we may further confirm UFT's role in early-stage lung cancer. Furthermore, wedge resection was done for older patients or patients with poor pulmonary function, and in these patients, hilar lymph nodes were not adequately sampled. Occult metastasis may have occurred in these patients. Despite these shortcomings, this study may serve as a cornerstone regarding failure patterns and risk factor analysis in stage I NSCLC patients.

## CONCLUSION

Curative surgery remains the major treatment for stage I patients according to the current National Comprehensive Cancer Network guidelines.^2^ However, with recurrence rates of 20% to 50% among resected early-stage NSCLC patients.^18,19,26–29^ That is why we designed an effective prognostic model based on clinical, pathological, and surgical covariates. Further investigation might be necessary to define if higher risk groups may benefit from a different follow-up schedule or image study protocol and adjuvant treatment options.
