# Recurrence Risk Factors Analysis for Stage I Non-small CellLung Cancer: Erratum

**DOI:** 10.1097/01.md.0000472178.01382.79

**Published:** 2015-10-02

In the article “Recurrence Risk Factors Analysis for Stage I Non-small Cell Lung Cancer”,^1^ which appeared in Volume 94, Issue 32 of *Medicine*, the affiliations footnote contained several errors. The article has since been corrected online.
